# The *Arabidopsis* Lectin Receptor Kinase LecRK-I.8 Is Involved in Insect Egg Perception

**DOI:** 10.3389/fpls.2019.00623

**Published:** 2019-05-10

**Authors:** Caroline Gouhier-Darimont, Elia Stahl, Gaetan Glauser, Philippe Reymond

**Affiliations:** ^1^Department of Plant Molecular Biology, University of Lausanne, Lausanne, Switzerland; ^2^Neuchâtel Platform of Analytical Chemistry, University of Neuchâtel, Neuchâtel, Switzerland

**Keywords:** *Arabidopsis thaliana*, lectin-like receptor kinase, oviposition, *Pieris brassicae*, *PR1* expression, herbivory

## Abstract

Plants induce defense responses after insect egg deposition, but very little is known about the perception mechanisms. In *Arabidopsis thaliana*, eggs of the specialist insect *Pieris brassicae* trigger accumulation of reactive oxygen species (ROS) and salicylic acid (SA), followed by induction of defense genes and localized necrosis. Here, the involvement of the clade I L-type lectin receptor kinase LecRK-I.8 in these responses was studied. Expression of *LecRK-I.8* was upregulated at the site of *P. brassicae* oviposition and egg extract (EE) treatment. ROS, SA, cell death, and expression of *PR1* were substantially reduced in the *Arabidopsis* knock-out mutant *lecrk-I.8* after EE treatment. In addition, EE-induced systemic resistance against *Pseudomonas syringae* was abolished in *lecrk-I.8*. Expression of ten clade I homologs of *LecRK-I.8* was also induced by EE treatment, but single mutants displayed only weak alteration of EE-induced *PR1* expression. These results demonstrate that LecRK-I.8 is an early component of egg perception.

## Introduction

Herbivorous insects often deposit eggs on leaves and these seemingly inert structures have been shown to induce defense responses in different plant species (Reymond, 2013; Hilker and Fatouros, 2015). For example, direct defenses include localized hypersensitive response (HR)-like necrosis (Shapiro and DeVay, 1987; Balbyshev and Lorenzen, 1997; Fatouros et al., 2014; Griese et al., 2017), neoplasm formation (Doss et al., 2000; Petzold-Maxwell et al., 2011), production of ovicidal substances (Seino et al., 1996; Geuss et al., 2017), or tissue crushing (Desurmont et al., 2011), which all impair egg attachment or survival. In addition, oviposition-induced production of volatiles provides indirect defense by attracting egg parasitoids (Hilker et al., 2002; Fatouros et al., 2008; Büchel et al., 2011; Tamiru et al., 2011). Besides impacting egg survival, induced responses may also affect future success of hatching larvae. Indeed, reduced performance of larvae feeding on oviposited plants has been observed in pine (Beyaert et al., 2012), elm (Austel et al., 2016), *Nicotiana attenuate* (Bandoly et al., 2015, 2016), and Brassicaceae species (Pashalidou et al., 2012; Geiselhardt et al., 2013; Bonnet et al., 2017; Lortzing et al., 2019). However, this effect was not found with all tested insects and even an increased performance of a generalist insect feeding was reported in *Arabidopsis* (Bruessow et al., 2010; Pashalidou et al., 2012; Bandoly et al., 2016). Also, oviposition diminishes infection by bacterial pathogens, presumably for the benefit of hatching larvae (Hilfiker et al., 2014).

Although it is now clearly established that plants respond to oviposition, information on the nature of egg-associated cues that trigger the observed changes is scarce (Hilker and Fatouros, 2015). Bruchins are long-chain α,ω-diols purified from female bruchid beetles. They stimulate neoplasm formation on pea pods (Doss et al., 2000). Extracts from the female planthopper *Sogatella furcifera* contain various phospholipids that induce production of the ovicidal substance benzyl benzoate in Japonica rice varieties (Seino et al., 1996; Yang et al., 2013). Benzyl cyanide is found in accessory reproductive glands from *Pieris brassicae* and induces leaf chemical changes that arrest an egg parasitoid on *Brassica oleracea* (Fatouros et al., 2008). Unknown proteins from oviduct secretions of the elm leaf beetle and the pine sawfly are responsible for egg-induced volatile emission (Meiners and Hilker, 2000; Hilker et al., 2005). Besides elicitors in secretions that are probably coating the egg surface, active molecules are also present within the egg. Crushed egg extract (EE) triggers neoplasm formation in pea (Doss et al., 1995) and arrest of parasitoids in maize (Salerno et al., 2013). EE from *P. brassicae* induces HR-like and expression of defense genes in *Arabidopsis* and *Brassica nigra* (Little et al., 2007; Bonnet et al., 2017). The activity is not proteinaceous and is enriched in the lipid fraction but a precise chemical characterization is still lacking (Bruessow et al., 2010; Gouhier-Darimont et al., 2013). Data thus indicate that various external and internal egg compounds activate defenses but how they reach a putative plant perception machinery is currently unknown.

The signal transduction pathway that links oviposition to downstream defense responses is starting to be unveiled. Reactive oxygen species (ROS) can be detected in oviposited or EE-treated plants, at the site of treatment (Little et al., 2007; Gouhier-Darimont et al., 2013; Bittner et al., 2017; Geuss et al., 2017). Salicylic acid (SA), a known signaling molecule in defense against biotroph pathogens, accumulates to high levels in response to insect eggs or EE in different plants, suggesting that the SA pathway is involved (Bruessow et al., 2010; Bonnet et al., 2017; Geuss et al., 2017; Lortzing et al., 2019). Indeed, the SA-responsive gene *PR1* is induced by oviposition (Little et al., 2007; Fatouros et al., 2014; Geuss et al., 2017) and its expression is abolished in SA-signaling *Arabidopsis* mutants *eds1-2*, *sid2-1*, and *npr1-1* (Gouhier-Darimont et al., 2013). EE-triggered *PR1* induction also depends on ROS accumulation but the nature of the ROS-generating process is still unknown, since *PR1* induction is still observed in mutants of NADPH oxidases (*rbohD/F*) that participate in pathogen-induced ROS production (Gouhier-Darimont et al., 2013). Ultimately, oviposition triggers a transcriptome signature that involves expression of many stress- and defense-related genes, and which is similar to SA-related transcriptomic responses to pathogens (Little et al., 2007; Fatouros et al., 2008; Büchel et al., 2011; Geuss et al., 2017; Drok et al., 2018). Furthermore, eggs from distantly related insect species induce the same defense genes, suggesting a common signaling pathway (Bruessow et al., 2010). Collectively, these findings are strikingly similar to the detection of pathogen-associated molecular patterns (PAMPs) by the plant innate immune system, a process called pattern-triggered immunity (PTI) (Boller and Felix, 2009).

During plant pathogenesis, bacterial or fungal PAMPs are recognized by cell-surface pattern recognition receptors (PRRs) that constitute a large group of conserved proteins. These PRRs are receptor-like proteins (RLPs) or receptor-like kinases (RLKs) that share a transmembrane domain and a highly variable extracellular domain responsible for the specific binding of PAMPs. In addition, RLKs possess a cytosolic kinase domain (Boutrot and Zipfel, 2017). In *Arabidopsis*, hundreds of genes encode RLKs, and RLPs (Shiu et al., 2004), but only a handful of PRRs have been characterized, including the well-known flagellin and chitin receptors FLS2 and CERK1, respectively (Boutrot and Zipfel, 2017). To date, no PRR for an egg-associated elicitor has been identified. Previously, searching for RLKs that may be related to egg recognition in *Arabidopsis*, we discovered that a lectin receptor kinase, LecRK-I.8, was involved in the response to *P. brassicae* EE. *LecRK-I.8* was upregulated by oviposition and EE-treatment, and a T-DNA knock-out line exhibited a drastic reduction of EE-induced *PR1* expression (Little et al., 2007; Gouhier-Darimont et al., 2013). LecRK-I.8 is a L- (legume) type LecRK, whose family members have been associated with plant immunity (Singh and Zimmerli, 2013; Wang and Bouwmeester, 2017), and belongs to a subclade of eleven closely related members (Bellande et al., 2017). Here, we further investigated the role of *LecRK-I.8* and its homologs in *Arabidopsis* responses to *P. brassicae* eggs.

## Materials and Methods

### Plant and Insect Material, Pathogens, and Growth Conditions

*Arabidopsis thaliana* Col-0 and mutant plants were grown in a growth chamber (Reymond et al., 2004) and were 4–5 week-old at the time of treatments. The *lecrk-I.8* T-DNA (SALK_066416) mutant was described in Gouhier-Darimont et al. (2013). For other *lecrk* mutants, T-DNA insertion lines were obtained from the Nottingham *Arabidopsis* Stock Center. Specific forward and reverse primers were designed with SIGnAL T-DNA verification tool for all lines^1^. T-DNA lines and primers are listed in Supplementary Table S1.

A colony of *P. brassicae* was reared on *B. oleracea* var. *gemmifera* in a greenhouse (Bonnet et al., 2017). *Spodoptera littoralis* eggs were obtained from Syngenta (Stein, Switzerland).

### Cloning and Plant Transformation

For pLecRK-I.8::NLS-GFP-GUS reporter line, the *LecRK-I.8* promoter (795 bp) was amplified with Phusion enzyme (New England Biolabs) using specific primers (Supplementary Table S1) and cloned into pDONRP4-P1r (Thermo Fisher Scientific) to produce the Entry clone. Using the LR CLonase II (Thermo Fisher Scientific), the entry clone was cloned in the destination vector pMK7S^∗^NFm14GW,0 (Karimi et al., 2007). Plants were transformed using the floral-dip method (Clough and Bent, 1998) and selected on ½ MS agar containing 50 μg/ml Kanamycin.

For complementation of *lecrk-I.8*, the *LecRK-I.8* promoter and coding sequence was amplified with Phusion enzyme (New England Biolabs) using specific primers (Supplementary Table S1). The *LecRK-I.8* amplicon (2769 bp) was cloned into a pGreenII0229-mVENUS plasmid containing the 3′ OCS terminator. Transformants were selected on ½ MS agar containing 40 μg/ml BASTA.

### Treatments

Egg extract preparation and application has been described previously (Bruessow et al., 2010; Gouhier-Darimont et al., 2013). In brief, *P. brassicae* eggs were crushed with a pestle in Eppendorf tubes. After centrifugation (15000 *g* for 3 min), the supernatant (EE) was stored at -20°C. Solid-phase extraction (SPE) was done as reported previously (Gouhier-Darimont et al., 2013). Total lipids were extracted with CHCl_3_/EtOH (1:1, v/v), the solution evaporated in a speedvac, and the dried material resuspended in 10% dimethylsulphoxide (DMSO). Lipids were then loaded on a Sep-Pak C18-reverse phase cartridge (Waters AG, Baden, Switzerland) and eluted with 50% MeOH, followed by 80% MeOH, and 100% MeOH. The 100% MeOH fraction (SPE-F) was dried under a nitrogen flux, and resuspended at a concentration of 5 μg/μl in 1% DMSO. For all experiments (except EE-induced SAR, see below), 2 μl of EE (equivalent to one egg batch of 20–30 eggs), or SPE fraction was deposited on the abaxial side of fully developped leaves. For flagellin treatment, a solution of 100 nM flg22 (Peptron.com) was infiltrated in three leaves of each of three plants and leaves were collected after 20 h. Water infiltration was used as control. For natural egg deposition, plants were placed in a tent containing *P. brassicae* butterflies for 2–4 h. Oviposited plants were then transferred to a growth chamber for 96 h.

### Histochemical Staining and SA Measurements

Reactive oxygen species visualization and quantification was done as in Gouhier-Darimont et al. (2013). GUS staining was done as in Little et al. (2007). Two leaves of each of six plants were treated with EE and 10–12 leaves were harvested after 72 h for ROS analysis. SA analysis was performed by ultra-high performance liquid chromatography-tandem mass spectrometry (UHPLC-MS/MS) as reported previously (Bruessow et al., 2010; Glauser et al., 2014). Three leaves of each of six plants were treated with EE. After 0, 48 and 96 h, 15 leaf discs of 10 mm diameter (ca. 100 mg FW) were collected, ground in liquid nitrogen, spiked with 10 μL of a 100 ng/mL solution of SA-d4 as internal standard, and extracted twice with a mixture of ethylacetate:formic acid (99.5:0.5, v/v). After evaporation, the dried residues were reconstituted in 100 μL of methanol 70%. An aliquot of 5 μL was injected in the UHPLC-MS/MS system (a 4000 QTRAP from ABSciex coupled to an Ultimate 3000 RS from Dionex). The mass spectrometer was operated in negative electrospray with the transitions *m/z* 137>93 and 141>97 for SA and SA-d4, respectively. Free SA quantification was achieved by internal calibration using 5 calibration points containing all SA-d4 at 10 ng/mL.

### Gene Expression Analysis

Two leaves of each of four plants were treated with EE. After 72 h, EE was carefully removed and leaf discs of 5 mm diameter were collected at the site of treatment. For each genotype, 6 leaf discs were used for RNA extraction and Quantitative RT-PCR analysis. Expression analysis of selected genes was described previously (Bruessow et al., 2010; Gouhier-Darimont et al., 2013). *SAND* (At2g28390) was used as a reference gene. The list of gene-specific primers can be found in Supplementary Table S1.

### EE-Induced SAR

SAR assay was performed as described previously (Hilfiker et al., 2014). *Pseudomonas syringae* pv. tomato DC3000 (*Pst*) was grown in King’s B medium containing 50 μg/ml rifampicin at 28°C. Overnight log phase cultures were washes three times with 10 mM MgCl_2_ and diluted to OD_600_ of 0.0005 for leaf inoculation. To induce SAR, three fully developped leaves of each of six Col-0 and *lecrk-I.8* plants were treated with 2 μl × 2 μl of EE from the abaxial side of the leaf. Five days after the treatment, EE was carefully removed with a brush and three untreated leaves distal to the site of EE treatment were inoculated with a suspension of *Pst* at OD_600_ 0.0005 in 10 mM MgCl_2_ from the abaxial side with a 1 ml needleless syringe. The same amount of untreated plants was inoculated with *Pst* and served as controls. Growth of *Pst* in inoculated leaves was measured 48 h later by serial dilutions on LB plates.

## Results

### Insect Eggs Trigger Local Expression of *LecRK-I.8*

**FIGURE 1:**
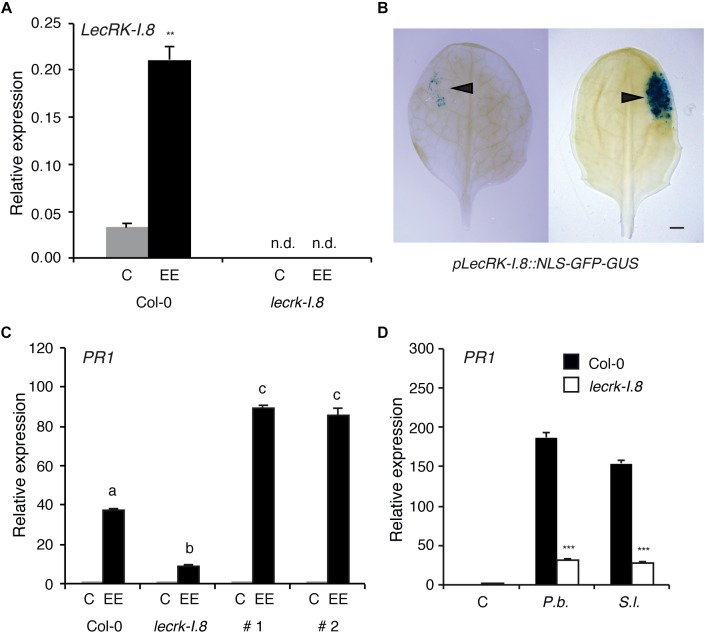
LecRK-I.8 is involved in *Arabidopsis* response to insect eggs. **(A)** *LecRK-I.8* expression 72 h after application of *P. brassicae* egg extract (EE) in Col-0 and *lecrk-I.8* T-DNA mutant. Untreated plants were used as control (C). Significant difference between control and treatment is indicated (Student’s *t*-test, ^∗∗^*P* < 0.01). n.d., not detected. Mean ± SE of three technical replicates are shown. This experiment was repeated twice with similar results. **(B)** Natural deposition of *P. brassicae* eggs (left) or application of 2 μl of *P. brassicae* EE (right) onto a leaf of pLecRK-I.8::NLS-GFP-GUS line. GUS expression was analyzed by histochemical staining 96 h after treatment. Arrowheads indicate the site of oviposition and EE application. Bar = 1 mm **(C)** *PR1* expression 72 h after *P. brassicae* EE treatment. #1 and #2 are two independent lines where *lecrk-I.8* was complemented with a LecRK-I.8-Venus construct. Different letters indicate significant differences (ANOVA followed by Tukey’s honest significant difference test, *P* < 0.05). Mean ± SE of three technical replicates are shown. This experiment were repeated once with similar results. **(D)** *PR1* expression 72 h after treatment with EE from *Pieris brassicae* (*P.b.*) or *Spodoptera littoralis* (*S.l.*) in Col-0 (black bars) and *leckrk-I.8* (white bars). Untreated plants were used as control (C). Significant differences between control and treatment are indicated (Student’s *t*-test, ^∗∗∗^*P* < 0.001). Mean ± SE of three technical replicates are shown. This experiments were repeated twice with similar results.

Expression of *LecRK-I.8* (At5g60280) in response to *P. brassicae* EE treatment was monitored by QPCR and showed a more than fourfold increase 72 h after application (Figure 1A). A T-DNA knock-out line (*lecrk-I.8*, SALK_066416) had no detectable *LecRK-I.8* expression in presence or absence of EE, confirming the KO nature of this mutant (Figure 1A). Using a promoter-NLS-GFP-GUS reporter line, we observed a strong activation of *LecRK-I.8* expression at the site of natural *P. brassicae* oviposition or at the site of EE treatment, indicating a precisely localized activation of this RLK (Figure 1B). As reported previously (Gouhier-Darimont et al., 2013), *P. brassicae* EE treatment triggered a substantial induction of the SA-marker gene *PR1*, and this response was significantly, although not fully, reduced in the *lecrk-I.8* mutant (Figure 1C). Similarly, induction of egg-responsive *CHIT*, *TI*, and *SAG13* (Little et al., 2007) was lower in *lecrk-I.8* (Supplementary Figure S1). To demonstrate that LecRK-I.8 was directly responsible for the reduced expression of *PR1*, we generated *Arabidopsis* transgenic lines where *lecrk-I.8* was complemented with the *LecRK-I.8* gene under the control of its own promoter. In two independent lines, EE-dependent *PR1* induction was restored to even higher levels than WT plants (Figure 1C). Finally, *PR1* induction in response to EE from *P. brassicae* or *S. littoralis* was similarly, reduced in *lecrk-I.8*, indicating that perception of eggs from two widely divergent herbivore species may depend on the same RLK (Figure 1D).

### LecRK-I.8 Modulates EE-Induced ROS and Cell Death

**FIGURE 2:**
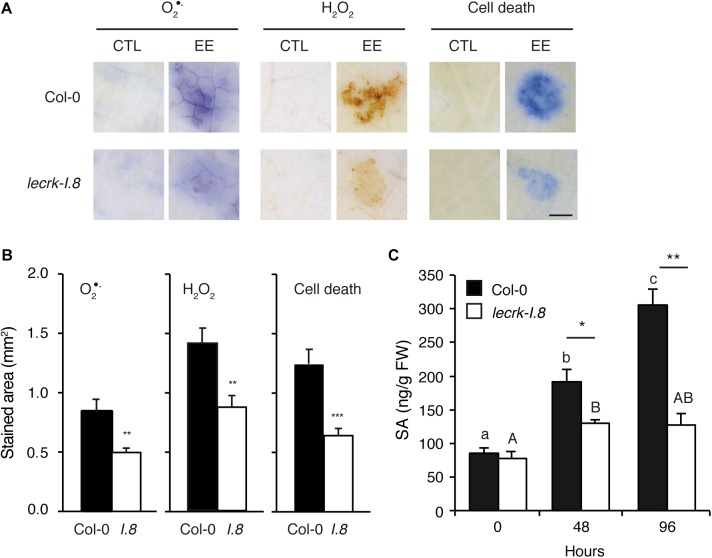
LecRK-I.8 is involved in signaling of *Arabidopsis* response to EE. **(A)** Leaves from Col-0 and *leckrk-I.8* were treated with *P. brassicae* EE for 72 h. Histochemical staining of leaves with nitroblue tetrazolium (NBT) to detect O_2_^∙-^, 3,3-diaminobenzidine (DAB) to detect H_2_O_2_, and trypan blue to detect cell death was performed. Untreated plants were used as controls (CTL). Panels are close-up images of the spotted area. Representative photographs from several replicates are shown. Bar = 1 mm. **(B)** Quantification of ROS and cell death accumulation in response to EE treatment as in **(A)**. Stained area was measured on images with ImageJ software (*n* = 10). Means ± SE are shown. Significant differences are indicated (Student’s *t*-test, ^∗∗^*P* < 0.01). *I.8*, *lecrk-I.8*. **(C)** Free salicylic acid (SA) was quantified in leaf discs of 10 mm diameter (*n* = 15) during 96 h after application of *P. brassicae* EE in Col-0 (black bars) and *lecrk-I.8* (white bars). Means ± SE of three independent biological replicates are shown. Different letters indicate significant differences (ANOVA followed by Tukey’s honest significant difference test, *P* < 0.05). Significant difference between wild-type and mutant are indicated (Student’s *t*-test, ^∗^*P* < 0.05, ^∗∗^*P* < 0.01, ^∗∗∗^*P* < 0.001).

Oviposition triggers local ROS accumulation and cell death that depend on an intact SA pathway (Little et al., 2007; Gouhier-Darimont et al., 2013). We quantified O_2_^∙-^ and H_2_O_2_, as well as cell death, in plants treated with EE for 72 h. Local accumulation of ROS and cell death was significantly reduced in *lecrk-I.8* compared to Col-0, implying that LecRK-I.8 plays an important role in this response (Figure 2A,B). However, the mutant exhibited ca. 50% of the wild-type response to EE treatment, suggesting that other factors participate in ROS or cell death accumulation.

*Pieris brassicae* eggs or EE treatment induce a strong SA accumulation (Bruessow et al., 2010). We monitored free SA levels in Col-0 and *lecrk-I.8* from 0 to 4 days after EE treatment. At the start of the treatment, both genotypes had similar constitutive SA levels. However, the gradual EE-dependent increase of SA found in Col-0 was severely impaired in the mutant, although levels after 2 days of EE treatment were significantly higher than at time 0, indicating that a residual amount of SA can still accumulate in *lecrk-I.8* (Figure 2C). These results show that LecRK-I.8 is the main component controlling EE-induced SA accumulation.

**FIGURE 3:**
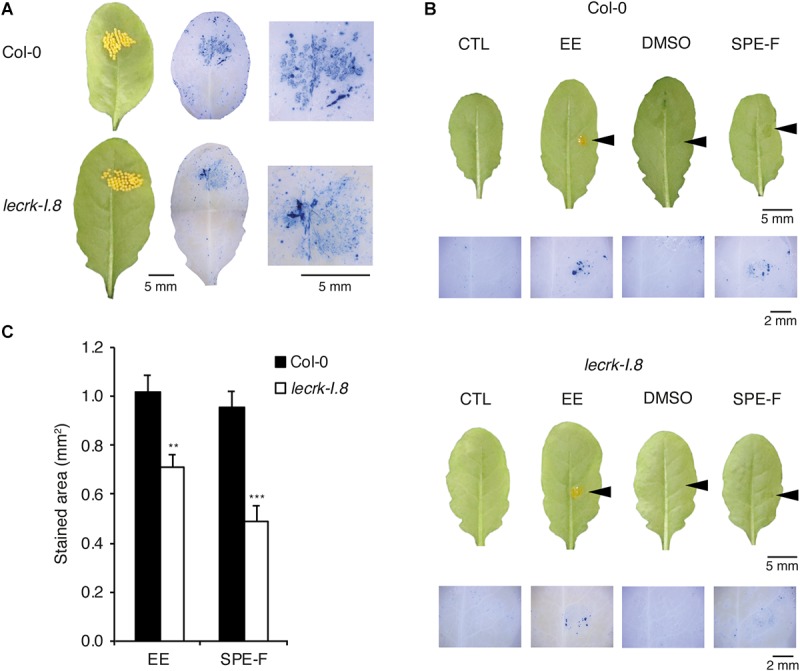
Induction of cell death in response to natural oviposition, EE, and purified egg lipids. **(A)** Trypan blue staining to detect cell death was performed on *P. brassicae* oviposited Col-0 and *lecrk-I.8* plants. Butterflies were allowed to lay eggs for 2 h on the plants and trypan blue staining was performed 72 h later. Representative leaves before and after staining and close-up images of the oviposited sites are shown. **(B)** Leaves from Col-0 and *lecrk-I.8* were treated with 2 μl of *P. brassicae* EE, or with 2 μl of a 5 μg/μl solution of a solid phase extraction fraction of total egg lipids eluted with 100% MeOH (SPE-F). Untreated plants (CTL) and plants treated with 1% DMSO served as controls. Arrowheads indicate the site of treatment. Cell death was visualized 72 h after treatments by trypan blue staining. Panels are close-up images of the treated area. **(C)** Quantification of cell death in Col-0 and *lecrk-I.8* in response to EE and SPE-F as in **(B)**. Stained area was measured on images with ImageJ software (*n* = 12). Means ± SE are shown. Significant difference between wild-type and mutant are indicated (Student’s *t*-test, ^∗∗^*P* < 0.01, ^∗∗∗^*P* < 0.001).

We showed previously that total *P. brassicae* egg lipids and a lipidic fraction eluted with 100% MeOH from a SPE strongly activated *PR1* expression (Gouhier-Darimont et al., 2013). To test the specificity of LecRK-I.8 in response to active egg components, we monitored cell death in naturally oviposited leaves and in leaves treated with EE or with the SPE fraction. Localized cell death was triggered by all treatments and significantly reduced in *lecrk-I.8* compared to Col-0 (Figure 3).

**FIGURE 4:**
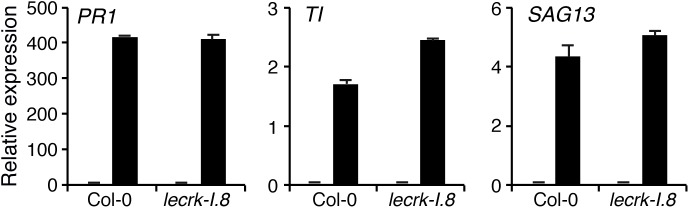
LecRK-I.8 is not involved in flagellin perception. Expression of EE-inducible genes was monitored after infiltration of 100 nM flg22 for 20 h (black bars). Plants infiltrated with water were used as control (gray bars). Means ± SE of three technical replicates are shown. This experiment was repeated twice with similar results.

Because responses triggered by insect eggs resemble those induced during PTI, we assessed the role of LecRK-I.8 in PAMP-induced gene expression. After infiltration of the known PAMP flagellin (flg22), expression of *PR1*, *CHIT*, and *SAG13* was significantly induced in Col-0 but also to a similar extent in *lecrk-I.8*, suggesting that LecRK-I.8 is not required for flagellin perception but plays a specific role in egg perception (Figure 4).

### EE-Induced SAR Depends on LecRK-I.8

**FIGURE 5:**
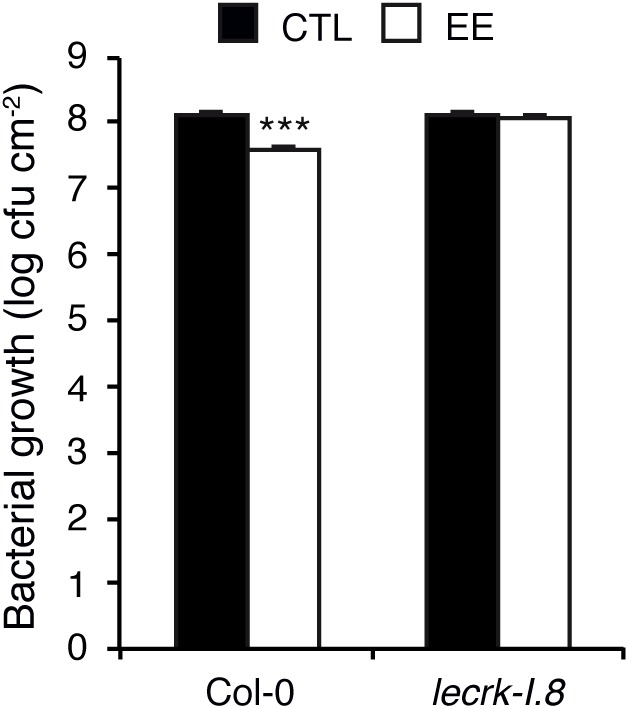
Egg extract-induced SAR depends on LecRK-I.8. Growth of *Pseudomonas syringae* pv. *tomato* DC3000 was monitored in distal (white bars) leaves after application of *P. brassicae* EE on local leaves for 5 days. Control plants (black bars) were only infected with bacteria. Means ± SE of three independent biological replicates are shown. Significant difference between control and treated plants is indicated (linear mixed model, ^∗∗∗^*P* < 0.001).

We previously found that oviposition by *P. brassicae* triggers a systemic acquired resistance (SAR) against the hemibiotroph bacterial pathogen *P. syringae* (Hilfiker et al., 2014). To investigate the role of LecRK-I.8 in egg-induced SAR, we pretreated three Arabidopsis leaves with *P. brassicae* EE, and after 5 days three distal leaves were inoculated with *P. syringae* pv. *tomato* DC3000 (*Pst*). After 2 days, bacterial growth was monitored, and compared to control plants not treated with EE. As reported previously, EE-pretreatment led to a significant inhibition of *Pst* growth in systemic leaves. Strikingly, this EE-induced SAR was abolished in *lecrk-I.8*, indicating that LecRK-I.8 is crucial for the establishment of EE-induced SAR (Figure 5).

### Role of *LecRK-I.8* Homologs

**FIGURE 6:**
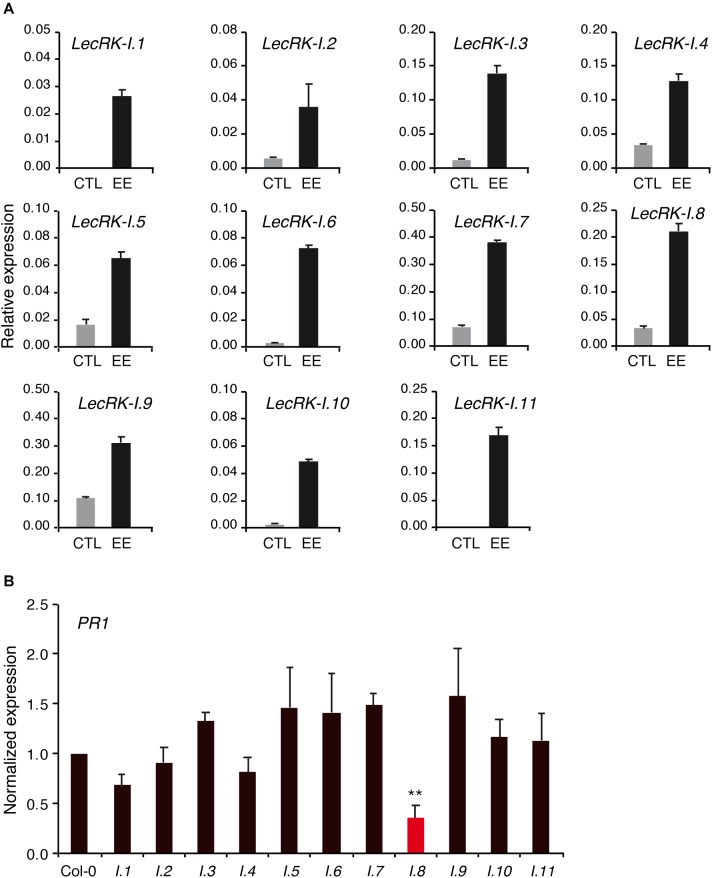
Role of *LecRK-I.8* homologs. **(A)** Expression of *LecRK-I.8* homologs 72 h after application of *P. brassicae* EE (black bars). Untreated plants were used as control (gray bars). Means ± SE of three technical replicates are shown. This experiment was repeated twice with similar results **(B)** *PR1* expression in *lecrk* mutants 72 h after application of *P. brassicae* EE. Values were normalized to Col-0. Means ± SE of three independent biological replicates are shown. Significant difference between Col-0 and each mutant is indicated (Student’s *t*-test, ^∗∗^*P* < 0.01).

LecRK-I.8 belongs to a subclade of 11 L-type LecRKs (Bellande et al., 2017). Since responses to EE tested in this study were not fully abolished in *lecrk-I.8*, we reasoned that this may be explained by some level of functional redundancy. We first assessed the expression of the 11 *LecRK-Is* in response to EE treatment. Like *LecRK-I.8*, all *LecRK-Is* genes were strongly up-regulated after 72 h of EE treatment (Figure 6A).

To investigate the role of each LecRK-Is in EE-induced gene expression, we obtained T-DNA mutants for all members, and quantitated *PR1* expression after EE treatment. Overall, none of the mutant except *lecrk-I.8* displayed a significantly altered *PR1* induction compared to Col-0, although there was a trend for reduced *PR1* induction in *lecrk-I.1* and *lecrk-I.4* (Figure 6B).

## Discussion

Plants are equipped with a perception system to detect the presence of insect eggs and induce the accumulation of diverse signaling molecules including ROS and SA, followed by the activation of defense genes and localized cell death. Currently, very few insect-derived cues have been characterized and no plant receptor is known. We show here that a knock-out of the L-type lectin receptor kinase LecRK-I.8 is impaired in *Arabidopsis* responses to insect eggs. Indeed, EE-induced accumulation of the early signals O_2_^-^ and H_2_O_2_, and of SA are significantly reduced in *leckrk-I.8*. In addition, expression of EE-inducible genes and localized cell death are also inhibited. These results indicate that LecRK-I.8 acts upstream of a signaling cascade that controls responses to oviposition. LecRK-I.8 is a plasma-membrane localized receptor kinase (Wang et al., 2017) and, as such, may well constitute a PRR for yet unknown egg-associated molecular patterns (EAMPs). Indeed, we show that a lipidic fraction from *P. brassicae* eggs triggers localized cell death and that this response is significantly attenuated in *lecrk-I.8*, suggesting that LecRK-I.8 is involved in the sensing of an egg-derived lipidic compound. Testing this hypothesis will require the chemical identification of *P. brassicae* EAMPs and binding studies with LecRK-I.8 produced in heterologous systems. Alternatively, LecRK-I.8 may function as a co-receptor to modulate the activity of EAMP potential PRR(s). Searching for LecRK-I.8 interacting partners may help answering this question. Furthermore, although *Arabidopsis* response to insect eggs share similarities with PTI, the finding that flg22-induced *PR1* expression is not affected in *lecrk-I.8* suggests that LecRK-I.8 plays a specific role and further supports the idea that it is involved in EAMP perception.

Interestingly, expression of *LecRK-I.8* and its homologs is induced by EE treatment and experiments with the LecRK-I.8::NLS-GFP-GUS reporter line indicate that this activation is highly localized, at the site of egg deposition or EE treatment. Induced expression of PRR genes in response to PAMP treatment has been previously observed (Zipfel et al., 2006) and could represent a way to enhance the plant’s ability to detect and respond to incoming pathogens. Here, the presence of eggs may as well stimulate a forward loop to increase the amount or number of potential LecRK receptors.

Generally, responses to oviposition in *Arabidopsis* have also been observed with EE treatment. Indeed, similar effects have been reported with both natural oviposition and EE treatment for defense gene expression, ROS production, cell death, SA accumulation, and EE-induced SAR (Little et al., 2007; Bruessow et al., 2010; Gouhier-Darimont et al., 2013; Hilfiker et al., 2014), strongly suggesting that EE treatment reflects natural oviposition. However, we cannot formally rule out that, in addition, intact eggs actively secrete elicitors or effectors that affect processes that have not yet been discovered. Capturing such molecules might be a challenge since eggs are firmly glued to the leaf surface. Current data indicate that passive diffusion of egg elicitors out of the egg into the leaf is the most parsimonious explanation for the observed responses. Once the exact chemical nature of the elicitor(s) will be obtained, further research should aim at understanding how they reach potential cell surface receptors.

Besides activating a signaling pathway that ultimately provokes an HR-like response and the expression of numerous defense genes, we previously reported that oviposition triggers a SAR that restricts bacterial growth in systemic leaves (Hilfiker et al., 2014). This phenomenon depends on a functional SA pathway and may constitute a strategy evolved by butterflies to protect the host on which eggs are deposited and will hatch (Hilfiker et al., 2014). Strikingly, we found here that EE-induced SAR is abolished in *lecrk-I.8*, in line with the lack of SA induction in the mutant. It thus appears that LecRK-I.8 is necessary for distinct responses to oviposition, confirming an involvement at the early phase of egg perception. Furthermore, the observation that the response to EE from two widely divergent insect species, *P. brassicae* and *S. littoralis*, is similarly impaired in *lecrk-I.8* strongly supports the notion that a generic EAMP is perceived by *Arabidopsis* and that this requires LecRK-I.8.

Although we demonstrate that LecRK-I.8 plays a significant role in *Arabidopsis* responses to eggs, expression of EE-inducible genes as well as ROS, SA, and cell death accumulation were not completely abolished in *lecrk-I.8*. At least two non-excluding hypotheses can explain these observations. First, plants contain a myriad of PRRs and specifically perceive different PAMPs from the same pathogen (Boutrot and Zipfel, 2017). It is conceivable that insect eggs release several EAMPs and that LecRK-I.8 is only perceiving one of them. As we are currently lacking a purified EAMP from *P. brassicae* eggs, we use a crude EE that may contain more than one active molecules. Second, all closely related homologs of *LecRK-I.8* were induced by EE treatment, implying a role in perception. Although single mutants, except *lecrk-I.8*, are barely affected in EE-induced *PR1* expression, we cannot exclude some level of redundancy that may contribute to the residual responses in *lecrk-I.8*. Unfortunately, *LecRK-I.8* homologs are clustered in two loci of the *Arabidopsis* genome (Supplementary Figure S2), rendering the generation of higher order mutants by crossing difficult. Generating large deletions of *LecRK-Is* clusters by CRISPR-Cas9 technology may represent a useful strategy to test the role of these receptors in the responses to insect eggs.

Intriguingly, LecRK-I.8 was recently identified as a potential sensor for extracellular NAD^+^ in *Arabidopsis* (Wang et al., 2017). Besides its role as an intracellular redox carrier that controls multiple metabolic reactions, including some defenses processes (Pétriacq et al., 2016), NAD(P) can be found in extracellular spaces after wounding or during pathogenesis (Zhang and Mou, 2009). Furthermore, exogenous application of NAD(P) triggers the expression of defense genes, including *PR1*, suggesting that perception of this extracellular signal could reinforce plant defenses (Zhang and Mou, 2009). Indeed, there is growing evidence that passive release of metabolites upon cell damage modulates innate immunity (Gust et al., 2017). Although the concentration of exogenous NAD^+^ needed to trigger responses (millimolar range) is much higher than the binding affinity of LecRK-I.8 to NAD^+^ (nanomolar range) (Wang et al., 2017), this finding raises the question of whether NAD^+^ is involved in insect egg perception. Preliminary purification of *P. brassicae* EE has indicated that the active EAMP is present in a lipidic fraction that is unlikely to contain NAD^+^ (Bruessow et al., 2010; Gouhier-Darimont et al., 2013). In addition, we show here that LecRK-I.8 is involved in the response to this lipidic fraction. Egg EAMP(s) could however trigger the release of extracellular NAD^+^, which would then be perceived by LecRK-I.8. Alternatively, we cannot formally exclude that LecRK-I.8 binds two different ligands. Future experiments should aim at clarifying these open questions.

Recent years have seen an emergence of studies implying LecRKs in plant immunity (Singh and Zimmerli, 2013; Wang and Bouwmeester, 2017). For instance, the closely related LecRK-I.9 mediates resistance to *Phytophthora brassicae* and *P. syringae* (Bouwmeester et al., 2011; Balagué et al., 2016). Interestingly, LecRK-I.9 was shown to bind extracellular ATP, in analogy with the NAD-binding property of LecRK-I.8 (Choi et al., 2014). Other members of clade I are also involved in defense against *Phytophthora* sp. or *Alternaria brassicicola* (Wang et al., 2014). LecRK-V.2, -V.5, -VI.2, -VII.1, and -IX.2 modulate PTI responses (Desclos-Theveniau et al., 2012; Singh et al., 2012; Luo et al., 2017; Yekondi et al., 2018). In rice, a cluster of three G-type LecRKs confers resistance to the phloem-sucking brown planthopper (Liu et al., 2015). The *Arabidopsis* B-type LecRK LORE recognizes a bacterial PAMP lipopolysaccharide (Ranf et al., 2015). However, information about how LecRKs function at the molecular level and whether they act as PRRs or modulators of PRR signaling complexes is still lacking.

In conclusion, we have identified an important component of *Arabidopsis* perception system for insect eggs. LecRK-I.8 plays a role in early signal transduction steps and controls several responses to *P. brassicae* eggs. Future studies should focus on identifying potential egg-derived ligands for LecRK-I.8 and investigating the occurrence of such ligand-receptor pair in other plant species, as well as in the context of different egg-plant interactions.

## Data Availability

All datasets for this study are included in the manuscript and/or the Supplementary Files.

## Author Contributions

CG-D, ES, and PR designed and carried-out the experiments. GG quantified the salicylic acid. PR wrote the manuscript with the help of all authors.

## Conflict of Interest Statement

The authors declare that the research was conducted in the absence of any commercial or financial relationships that could be construed as a potential conflict of interest.

## Footnotes

1 http://signal.salk.edu/tdnaprimers.2.html

## Supplementary Material

FIGURE S1 Expression of EE-inducible genes in *lecrk-I.8*. Expression of *TI* (At1g73260), *CHIT* (At2g43570), and *SAG13* (At2g29350) was measured 72 h after application of *P. brassicae* EE (black bars). Untreated plants were used as control (gray bars). Means ± SE of three technical replicates are shown. Significant difference between wild-type and mutant are indicated (Student’s *t*-test, ^∗∗∗^*P* < 0.001). This experiment was repeated twice with similar results.

FIGURE S2 Position of *LecRK-I.8* homologs on *Arabidopsis* chromosomes 3 and 5.

TABLE S1 List of primers used in this study.
