# Supplementary figures and images for: The Arabidopsis Lectin Receptor Kinase LecRK-I.8 Is Involved in Insect Egg Perception
Source: Front Plant Sci. 2019 May 10;10:623. doi: 10.3389/fpls.2019.00623 (PMC6524003; doi:10.3389/fpls.2019.00623)

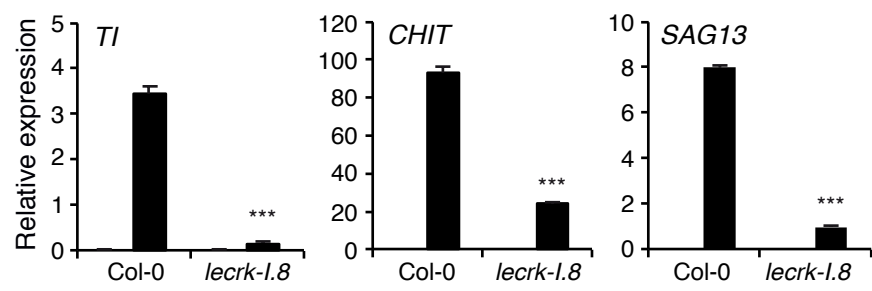

Figure S1

Supplement: FIGURE S1 — Expression of EE-inducible genes in lecrk-I.8. Expression of TI (At1g73260), CHIT (At2g43570), and SAG13 (At2g29350) was measured 72 h after application of P. brassicae EE (black bars). Untreated plants were used as control (gray bars). Means ± SE of three technical replicates are shown. Significant difference between wild-type and mutant are indicated (Student’s t-test, ∗∗∗P < 0.001). This experiment was repeated twice with similar results. [file Image_1.pdf]

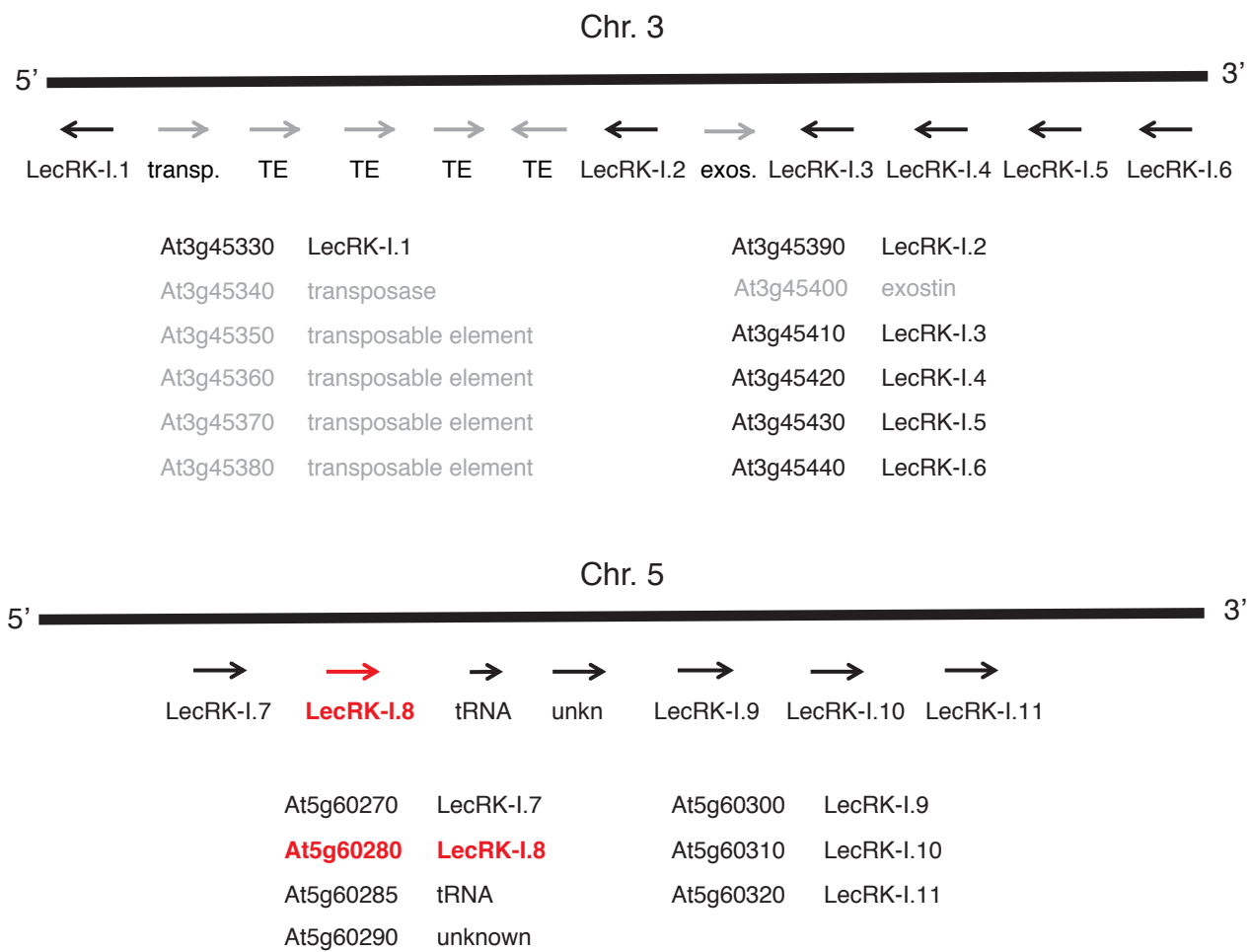

Figure S2

Supplement: FIGURE S2 — Position of LecRK-I.8 homologs on Arabidopsis chromosomes 3 and 5. [file Image_2.pdf]
