# Supplementary material for: The Arabidopsis Lectin Receptor Kinase LecRK-I.8 Is Involved in Insect Egg Perception
Source: Front Plant Sci. 2019 May 10;10:623. doi: 10.3389/fpls.2019.00623 (PMC6524003; doi:10.3389/fpls.2019.00623)
Supplement: TABLE S1 — List of primers used in this study. [file Table_1.pdf]

Table S1. Primer list

A T-DNA genotyping

Mutant	T-DNA line	LP primer	RP primer
lecrk-I.1 (At3g45330)	SALK_052123	GTTCCTGTTGACGTTAGCTCG	GTCTGTGGAATCTGCTTCAGC
lecrk-I.2 (At3g45390)	SAIL_847_F07	TCAGGCACTGGAAAAACTCTG	GAAGCAGAAACGACAGATTGG
lecrk-I.3 (At3g45410)	SALK_087804C	TGCCATCTTACTTTTCCAACG	ATAAGCAAGCCTCTTTCCAGC
lecrk-I.4 (At3g45420)	SALK_091901	TTTGTGGCAGAAGTCGTAACC	AAATCAATGCATTCGAACTGG
lecrk-I.5 (At3g45430)	GABI_777H06	CGCTCTGCCTGTTATACTTGC	ATTGGCTGAAGAAGAAGAGGC
lecrk-I.6 (At3g45440)	GABI_353G10	CTGGCCCCTCTTAAAATTCAG	ATCAGGCAGCTCTAAGCTTCC
lecrk-I.7 (At5g60270)	SAIL_230_F04	CAAATGGTGACAACAACTTGC	GGAGAGGAGGTTTTGGTGAAG
lecrk-I.8 (At5g60280)	SALK_066416	ACACCACAACCAACAGGTCTC	CAGAAGCTGTTTCTCAATCGG
lecrk-I.9 (At5g60300)	SALK_042209	CTGAATACTTGCGTCTCCTGC	CAGCTTGCGAGGTTATGATTC
lecrk-I.10 (At5g60310)	SALK_039426C	AGGATGAGGAACTTCAGGGAG	GCGTAGTTTCATTGGCTCAAG
lecrk-I.11 (At5g60320)	GABI_757B03	CAAAACCGCCTTTTCCTAAAC	TGCATTTTGAAACTCCAGAAAC

B QPCR

Gene	Fw primer	Rv primer
LecRK-I.1	CCCGGATCGAAAAGCATTCA	GTTTTCTCCGGTTTCTTGGG
LecRK-I.2	CCCGGATCGAAAAGCTTTTG	GTTCAGAGATGTCAAGGCTC
LecRK-I.3	TCCTAAGTTGGGAGCTGATG	ACCAACATGAGGCTTCTCCA
LecRK-I.4	TGAAACTTGGGTTGCTCTGC	ATGGTTCCACTGAAACTGGC
LecRK-I.5	TGCTGAGTTGAACGGTAGGT	CTCAACTTGCACCCCGAATT
LecRK-I.6	CCAATGGTAGCCTTGACCAA	GGGTCTTTCCCATGATCATG
LecRK-I.7	GAGTTCAATGGAAGGTTGGG	GTTTTGCTTCAGGTAAGCCG
LecRK-I.8	CTACCTCAGGAAGACATAGC	CTGCAGTATCCGAAAAGTGG
LecRK-I.9	TGGTGATGAAGGTGTGAAGC	AGAACCGGTTTCTGGTCATC
LecRK-I.10	CTCTCAAGTCAGCAAGAGAC	GAGCCATCTGATGATCGGAA
LecRK-I.11	GACTCATTTTGTGTGTGCGC	GCTCTCAACAGTATCGAGCT
PR1 (At2g14610)	GTGGGTTAGCGAGAAGGCTA	ACTTTGGCACA TCCGAGTCT
CHIT (At2g43570)	GGAGAGTACTGCGACACAGAGAAA	GGCAGGAACCTGGTCTTGAGCAA
TI (At1g73260)	CCTCGTGGTTGCTGGTCCAAA	CCTCTCACATAGTCTTGGACGAAA
SAG13 (At2g29350)	GTCGTGCATGTCAATGTTGG	CCAAGGACAAACAGAGTTCG
SAND (At2g28390)	AACTCTATGCAGCATTTGATCCACT	TGATTGCATATCTTTATCGCCATC

C Cloning

Reporter line (pLecRK-I.8::NLS-GFP-GUS)	
attB4-LecRKI8p	GGGGACAACTTTGTATAGAAAAGTTGTTAAGTTTCTAACAACTTCCCTCT
attB1r-LecRKI8p	GGGGACTGCTTTTTTGTACAAACTTGTTGTTAATGCAACAAAGTGTTTG
Complementation line (*lecrk-I.8*|pLecRK-I.8::LecRK-I.8-mVENUS)	
LecRKI8p-Fw	TCCCCCCGGGAAGTTTCTAACAACTTCCCTCTT
LecRKI8cds-Rv	GCGGGATCCTCGTCCAATTCCGTATTGAATCGA
